# Digital Health Transformation of Integrated Care in Europe: Overarching Analysis of 17 Integrated Care Programs

**DOI:** 10.2196/14956

**Published:** 2019-09-26

**Authors:** Erik Baltaxe, Thomas Czypionka, Markus Kraus, Miriam Reiss, Jan Erik Askildsen, Renata Grenkovic, Tord Skogedal Lindén, János György Pitter, Maureen Rutten-van Molken, Oscar Solans, Jonathan Stokes, Verena Struckmann, Josep Roca, Isaac Cano

**Affiliations:** 1 Hospital Clinic de Barcelona Institut d'Investigacions Biomèdiques August Pi i Sunyer Universitat de Barcelona Barcelona Spain; 2 Center for Biomedical Network Research in Respiratory Diseases Madrid Spain; 3 Institute for Advanced Studies Vienna Austria; 4 Department of Economics University of Bergen Bergen Norway; 5 Agency for Quality and Accreditation in Health Care and Social Welfare Zagreb Croatia; 6 Department of Social Science NORCE Norwegian Research Centre Bergen Norway; 7 Syreon Research Institute Budapest Hungary; 8 School of Health Policy and Management Erasmus University Rotterdam Rotterdam Netherlands; 9 Institute for Medical Technology Assessment Erasmus University Rotterdam Rotterdam Netherlands; 10 Oficina eSalut Departament de Salut Generalitat de Catalunya Barcelona Spain; 11 Health Organisation, Policy, and Economics Centre for Primary Care and Health Services Research University of Manchester Manchester United Kingdom; 12 Department of Health Care Management Technische Universität Berlin Berlin Germany

**Keywords:** program evaluation, chronic patients, eHealth, elderly, integrated care, social support, telemonitoring, information and communication technology

## Abstract

**Background:**

Digital health tools comprise a wide range of technologies to support health processes. The potential of these technologies to effectively support health care transformation is widely accepted. However, wide scale implementation is uneven among countries and regions. Identification of common factors facilitating and hampering the implementation process may be useful for future policy recommendations.

**Objective:**

The aim of this study was to analyze the implementation of digital health tools to support health care and social care services, as well as to facilitate the longitudinal assessment of these services, in 17 selected integrated chronic care (ICC) programs from 8 European countries.

**Methods:**

A program analysis based on thick descriptions—including document examinations and semistructured interviews with relevant stakeholders—of ICC programs in Austria, Croatia, Germany, Hungary, the Netherlands, Norway, Spain, and the United Kingdom was performed. A total of 233 stakeholders (ie, professionals, providers, patients, carers, and policymakers) were interviewed from November 2014 to September 2016. The overarching analysis focused on the use of digital health tools and program assessment strategies.

**Results:**

Supporting digital health tools are implemented in all countries, but different levels of maturity were observed among the programs. Only few ICC programs have well-established strategies for a comprehensive longitudinal assessment. There is a strong relationship between maturity of digital health and proper evaluation strategies of integrated care.

**Conclusions:**

Notwithstanding the heterogeneity of the results across countries, most programs aim to evolve toward a digital transformation of integrated care, including implementation of comprehensive assessment strategies. It is widely accepted that the evolution of digital health tools alongside clear policies toward their adoption will facilitate regional uptake and scale-up of services with embedded digital health tools.

## Introduction

### Background

Digital health (eHealth) tools have been proposed to improve access to health care services, enhance care co-ordination and integration, enable self-management, support decision-making, enable monitoring, perform risk analysis, and facilitate proactive interventions [1]. It is within this context that the European Commission has defined eHealth as follows:

The use of Information and Communication Technologies in health products, services and processes, combined with organizational change in health care systems and new skills, in order to improve health of citizens, efficiency and productivity in health care delivery, and the economic and social value of health [2]

The implementation of digital health tools has constituted an area of major research and innovation in the past years [1,4-7]. For example, the impact of electronic health records on health care quality has been explored by Campanella et al who showed improvement in health care quality in terms of guideline adherence and time efficiency while reducing medication errors [8]. In parallel, most countries have recognized the value of patient portals [9-11]. The use of health monitoring devices in the general population is also increasing, with a broad spectrum of sophistication, including examples like integration between artificial intelligence and monitoring in a single device (ie, cardiac rhythm analysis) [12]. Moreover, the use of open-source algorithms for subject-specific as well as population-based risk prediction has been reported [13,14]. Some of these technologies are already in use for the management of chronic multimorbid patients [15-20].

It is currently accepted that eHealth tools can be particularly useful to support Integrated Chronic Care (ICC) programs for patients with multimorbidity, that is, co-occurrence of 2 or more chronic disorders within 1 individual [21]. Insights from the ICARE4EU project [6] concluded that eHealth improves care integration and management processes, but the project identified that inadequate funding mechanisms, poor interoperability, and inadequate technological support represent major barriers for adoption of technologically supported ICC. In fact, it is acknowledged that the takeoff of digital health tools to support ICC is progressing rather slowly. Also, regulatory aspects are still a concern [22-24] to achieve a proper balance between preservation of individual privacy and the need for health data sharing [25], as well as the increasing demand for health data analytics.

### The Conceptual Framework

The Sustainable intEgrated care modeLs for multimorbidity: delivery, Financing, and performancE (SELFIE) Horizon 2020 project [26] aims to produce evidence and applicable policy advice on ICC programs for people with multimorbidity. Within the project aims, the SELFIE conceptual framework [27] was developed. It comprises 6 core components of integrated care systems adapted from the World Health Organization, namely: (1) *Service delivery*, (2) *Leadership and governance*, (3) *Workforce*, (4) *Financing*, (5) *Technologies and medical products*, and (6) *Information and research*.

This paper focuses on 2 elements out of the 6 components of the SELFIE conceptual framework [27], which refer to the enabling role of digital health tools (*Technologies and medical products*) and assessment of ICC programs (*Information and research*). For each of these 2 components, the 3 levels of the SELFIE conceptual framework (micro, meso, and macro) were taken into account. The micro level is where the individual with multimorbidity interacts with care professionals and informal caregivers. The meso level relates to the organizational level and the institutional setup of providers. Finally, the macro level includes legislations, governance, policies, and system-wide changes at the national and international level.

For *Technologies and medical products*, the SELFIE framework stresses the need for digital health tools to be widely available and user-friendly to provide robust support to the care processes. At a micro level, the use of technology (eg, electronic medical records [EMR) and patient portals) can be a facilitator of collaborative care if tailored to the needs of the patient with multimorbidity. At a meso level, a shared information system (eg, EMR including shared care plans) among multiple providers and care settings can greatly facilitate communication, person-centeredness, personalized care, and care co-ordination. Finally, at a macro level, nationwide and international policies that foster technological development and innovation most likely would benefit from both implementation and continuous assessment of ICC programs for multimorbidity.

For *Information and research*, the project stresses the successful use of collected data from digital health tools for a 3-fold objective, namely: (1) Population health management; (2) Enhanced subject-specific health care delivery; and (3) Comprehensive assessment of ICC programs. At a micro level, currently collected individual-level data (eg, patient journey record) can effectively be used in the care process for individual risk prediction. At a meso level, shared information systems may further be used for service selection both at individual and group level (eg, triage systems and clinical predictive modelling) to strengthen the evidence base of complex integrated care interventions, as well as to develop indicators particularly relevant for the care of patients with multimorbidity. Alongside data ownership at a meso level, privacy and data protection legislation is an important consideration at macro level.

### Aims

It is well accepted that the existence of an important gap between the way in which the role of digital tools is understood and the effective uptake of digital transformation by the different stakeholders in the health care systems at European level. To enable the real implementation and scale-up of the digital health tools with all their potential, we undertook this overarching analysis. Our study aims to synthesize the experiences, views, and opinions (including barriers and enabling factors) of the stakeholders and their impact on the care process, as well as the role and desirable future developments of digital health tools, to foster transformation of health care systems toward sustainability by enhancing management of patients with multimorbidity. A second aim is to characterize the different programs with respect to maturity of their supporting digital health tools and the level of assessment of the ICC program.

Here, we present the results of an overarching analysis of the Thick Descriptions [28] of 17 promising ICC programs selected by the SELFIE project across 8 European countries: Austria, Croatia, Germany, Hungary, the Netherlands, Norway, Spain, and the United Kingdom. Within the overarching analysis, we focus on the different aspects of implementation of digital health tools supporting services and facilitating assessment strategies. This will lead to future directions defining how digital support can contribute to scale-up and evaluation of integrated care services. These services should focus on patient-centered health care provision with dynamic evaluation of technology-enabled integrated care programs, without compromising patient privacy.

## Methods

### Study Design

To select the 17 ICC programs, each country participating in the SELFIE project [29] applied a search strategy using the findings from an international scoping review, national publications on previous and on-going programs and projects, and consultation with national experts and networks. Details on the process of selection of the programs, as well as the list of the 17 selected programs per country, are reported in Multimedia Appendix 1. The 17 programs were grouped into 4 categories: (1) population health management programs (n=5); (2) frail elderly programs (n=6); (3) programs for individuals at the end-of-life and oncology patients (n=3); and (4) programs for vulnerable individuals who face problems in multiple life domains, like health, housing, and financial problems (n=3).

### Procedure and Data Collection

The Thick Description, a qualitative empirical research method, was used in SELFIE to gain a deep understanding of the ICC programs from the different stakeholders’ point of view [28,30]. The method undertaken included two different approaches: (1) Study of a variety of documents about each of the 17 ICC programs (ie, official and contractual documents, documents related to past evaluations, and factsheets from each ICC); and (2) interviews conducted with all relevant stakeholders (ie, program managers, initiators, representative of sponsor or payer organizations, health care professionals, informal caregivers, and patients or patient representatives). As described earlier, we concentrate on the two information technology-related dimensions in this paper.

Each partner-country interviewers underwent specific training on how to conduct and analyze the semistructured interviews to ensure uniform procedures. A total of 233 stakeholders were interviewed from November 2014 to September 2016 (see Multimedia Appendix 2 for more detailed information on stakeholder composition per country). The interviews were audio recorded and transcribed verbatim from the audio file by either the interviewer or an independent research transcriber. The resulting transcripts were analyzed by members of the local SELFIE teams using Mayring qualitative content analysis [31]. The quotations which were used in the thick descriptions were edited into readable forms and translated into English. The transcripts were not returned to participants for correction.

When writing this manuscript, we adhered to the COnsolidated criteria for REporting Qualitative research [32]. All information retrieved from the document analysis (including the stakeholders´ interviews) was processed according to the country-specific ethics statement listed under the subheading: *Ethics Approval and Consent to Participate*. The Thick Descriptions of the 17 ICC programs studied can be found on the SELFIE website [33].

### Overarching Analysis

The first author did a thematic analysis [34] on the Thick Descriptions of the 2 components of the SELFIE conceptual framework referring to the enabling role of digital health tools (Technologies and medical products) and assessment strategies of the ICC programs (Information and research). He then discussed findings with all other coauthors. For each of these 2 components, this secondary analysis of the Thick Descriptions considered the 3 levels of the SELFIE conceptual framework: micro, meso, and macro. As detailed in Table 1, a 3-level grading system (+ to +++) was developed and used, under the criteria of the coauthors (JR and IC), to score maturity of the 17 ICC programs for each of the 2 components assessed in this research. Finally, the maturity of each of the 2 components of the SELFIE conceptual framework is summarized as the average of the maturity at micro, meso, and macro level.

**Table:** Summary of the maturity grading criteria at micro, meso, and macro level for technologies and medical products and for information and research.

Component			Grading
**Technologies and medical products**			
	**Digital health tools (micro)**		
		Electronic medical records—EMR	+
		Personal health records at program level	++
		Personal health records at regional level	+++
	**Organizational interoperability (meso)**		
		Health information exchange	+
		Shared EMR^a^	++
		Shared case management systems^b^	+++
	**Digital transformation policies (macro)**		
		Only addressing EMR	+
		Several initiatives at program level	++
		National and regional strategic plans	+++
**Information and research**			
	**Evaluation strategies (micro)**		
		Planned evaluation	+
		Partial assessment	++
		Full assessment with published papers	+++
	**Risk assessment (meso)**		
		Clinical knowledge or rule-based	+
		Clinical predictive modelling tools	++
		Multilevel predictive modelling tools^c^	+++
	**Research and innovation policies (macro)**		
		Incipient initiatives	+
		Consolidated programs	++
		Strong co-ordination with EU^d^ programs	+++

^a^Shared electronic medical records among health care providers.

^b^Shared case management systems among health care providers to support integrated care pathways.

^c^Predictive modelling tools that combine information from various data sources, for example, clinical, population-based, biological, and patient-reported.

^d^EU: European Union.

### Ethics Approval and Consent to Participate

Letters of Medical Ethics Approval of study protocols, questionnaires, and informed consent forms were sent and approved by the European Commission as a Deliverable of the SELFIE project.

Austria: Letter from Institute for Advanced Studies (IHS) declaring that ethical approval is not necessary for the evaluation of the two Austrian Integrated Care programs, October 3, 2017.

Croatia: Statement from the Agency for Quality and Accreditation in Health Care and Social Welfare declaring that the two evaluation studies are not within the scope of work of Croatian Central Ethics Committee, August 28, 2017, with reference to Official Gazette No 121/07 and No 25/15.

Germany, Gesundes Kinzigtal: Letter from the Ethical Committee, Technische Universität Berlin, declaring that the research is ethically acceptable (Ref: ST_02_20170620, August 15, 2017).

Germany, Casaplus: Letter from the Ethical Committee, Technische Universität Berlin, declaring that the research is ethically acceptable (Ref: ST_01_20170428, August 4, 2017).

Hungary, OnkoNetwork: The research plan has been authorized under approval No IG/03092–000/2016 by the Director General of Moritz Kaposi General Hospital, based on the positive opinion of the Institutional Research Committee and the responsible person of the Hospital for data protection. The Institutional Ethics Committee of the hospital double-checked the research and publication plan and confirmed that no ethical concerns were emerging related to this research and to the publication of findings (October 10, 2018, Ref: IKEB_IG_04125-000_2018).

Hungary, Palliative Care Consult Service: Letter from the Medical Research Council (Tudomanyos es Kutatasetikai Bizottsag, ETT TUKEB) declaring that the research is granted with Professional-Ethical Approval (Ref: 18632–4/2017/EKU, 24–4-2017).

The Netherlands, Proactive Primary Care Approach for Frail Elderly (U-PROFIT): Letter from the Medical Ethical Committee (MEC) Erasmus Medical Center Rotterdam declaring that the research is exempt from the Medical Research Involving Human Subjects Act (Dutch acronym: WMO; Ref: MEC-2017-402, July 25, 2017).

The Netherlands, Care Chain Frail Elderly (CCFE): Letter from the Medical Ethical Committee (MEC) Erasmus Medical Center Rotterdam declaring that the research is exempt from the Medical Research Involving Human Subjects Act (Dutch acronym: WMO; Ref: MEC-2014.558, December 18, 2014).

The Netherlands, Better Together in Amsterdam North (BSiN): Letter from the Medical Ethical Committee (MEC) of the Free University Medical Centre declaring that the research is exempt from the Medical Research Involving Human Subjects Act (Dutch acronym: WMO; Ref: MEC-2017-121, March 10, 2017).

Norway, Learning Network for Whole, Co-ordinated and Safe Pathways: Letter from the Regional Committees for Medical and Health Research Ethics-West (Komité for medisinsk og helsefaglig forskningsetikk -REK vest) declaring that the research is ethically approved (Ref: 2017/632/REK vest, March 28, 2017).

Norway, Medically Assisted Rehabilitation Bergen: Letter from The Regional Committees for Medical and Health Research Ethics-West (Komité formedisinsk og helsefaglig forskningsetikk -REK vest) declaring that the research is ethically approved (2017/944/REK vest, June 21, 2017).

Spain, Barcelona-Esquerra (AISBE): Letter from Clinic Research Ethical Committee (Comitè Ètic d’Investigació Clinica—CEIC) of the Clinic Hospital of Barcelona (Ref: CIF-G-08431173, Reg. HCB 2017/0451, June 14, 2017).

Spain, Badalona Serveis Assistencials (BSA): Letter from Clinic Research Ethical Committee (Comitè Ètic d’Investigació Clinica—CEIC) of the Clinic Hospital of Barcelona (Ref: CIF-G-08431173, Reg. HCB 2017/0453, June 14, 2017).

United Kingdom: Approval was granted by the National Health Service (NHS) Health Research Authority Research Ethics Committee (SELFIE REF: 16/WM/0295; CLASSIC REF: 14/NW/0206, June 23, 2016).

All participants provided written informed consent before participation.

## Results

### Overview

Multimedia Appendix 3 provides a high-level description of results for the 2 components of the SELFIE conceptual framework considered in this study. In this table, main features of each ICC program (third and fourth columns) are provided per type of ICC program (first column) and country (second column). Extended results for the 17 ICC programs are reported in the text below. As a summary of the results, Table 2 displays an average of the 3-level maturity grading criteria stated in Table 1 for each of the 17 ICC programs, according to the micro, meso, and macro levels of the SELFIE conceptual framework.

### Technologies and Medical Products

The overarching analysis provided the following valuable insights on the implementation and exploitation of digital health tools to support ICC.

#### Digital Health Tools (Micro)

All 17 ICC programs have at least partial implementation of *EMR* and they are planning to enhance implementation of EMR in the future.

However, *specific personal health records* to enhance patient engagement are not considered in programs like Health Network Tennengau (HNT), Casaplus, OnkoNetwork (ON), Palliative Care Consulting Service (PCSS), Better together in Amsterdam North (BSiN), and Medically Assisted Rehabilitation (MAR). In such programs, digital information exchange between care provider and patient are either not considered or telephone is still the dominant tool for communications. Nevertheless, the use of personal health records has been key to support various telemonitoring services for patient self-management in programs like in GeroS (eKarton). Likewise, South Somerset Symphony Program (SSSP) and Salford Integrated Care Program (SICP), both from the United Kingdom, stress the role of digital health tools (ie, Patients Know Best) to support telemonitoring, albeit suffering from some implementation problems:

Tele-dermatology and we’re piloting it [...] the GP will take a photograph and email it and get a decision, they’re not doing suspected cancers obviously, but rashes. Yeah, we’ve done it

IP11_1—SICP

We've also got telehealth, that support. So we've got patients who are on telehealth in their homes, and each morning, the intensivists review the telehealth and see if there's any flags, like, if somebody is on [...] I'm trying to think. If somebody is on some sort of medication that they need to, you know, where fluid balance is an issue, if they've lost six pounds in weight that might flag some medication change. So they get them to weigh themselves, do their blood pressure, and so on. So, telehealth has been hugely supportive, actually, at keeping patients at home

IP08_2—SSSP

**Table 2:** Average maturity levels of the 17 Integrated Chronic Care programs.

Country	Program	Technologies and medical products	Information and research
Austria	HNT^a^	**++**	+
Austria	SMC^b^	**+**	+
Germany	Casaplus	**++**	++
Germany	GK^c^	**++**	++
Spain	AISBE^d^	**+++**	++
Spain	BSA^e^	**++**	++
Croatia	GeroS	**++**	+
Croatia	PCS^f^	**+**	+
Hungary	ON^g^	**+**	+
Hungary	PCCS^h^	**+**	+
The Netherlands	BSiN^i^	**++**	++
The Netherlands	CCFE^j^	**++**	++
The Netherlands	U-PROFIT^k^	**++**	++
Norway	LN^l^	**+**	+
Norway	MAR^m^	**+**	++
United Kingdom	SICP^n^	**++**	++
United Kingdom	SSSP^o^	**++**	+

^a^HNT: Health Network Tennengau.

^b^SMC: Sociomedical Centre Liebenau.

^c^GK: Gesundes Kinzigtal.

^d^AISBE: Area Integral de Salut Barcelona-Esquerra.

^e^BSA: Badalona Serveis Assistencials.

^f^PCS: Palliative Care System.

^g^ON: OnkoNetwork.

^h^PCCS: Palliative Care Consulting Service.

^i^BSiN: Better together in Amsterdam North.

^j^CCFE: Care Chain Frail Elderly.

^k^U-PROFIT: Proactive Primary Care Approach for Frail Elderly.

^l^LN: learning network.

^m^MAR: Medically Assisted Rehabilitation.

^n^SICP: Salford Integrated Care Program.

^o^SSSP: South Somerset Symphony Program.

It is of note that the availability of *personal health records at regional level*, which is the case with the programs Area Integral de Salut Barcelona-Esquerra (AISBE) and Badalona Serveis Assistencials (BSA) (La Meva Salut) [35], generates additional potential to foster collaborative work at micro level.

#### Organizational Interoperability (Meso)

Most of the programs use secure networks for *health information exchange* between hospitals and general practitioners, but with a broad spectrum of maturity. For example, the Casaplus program implemented a specific Web-based platform to support regular communication between case managers and nursing professionals only, but not primary care and the hospital. On the other hand, the health information exchange network used in HNT function only 1 way (Hospital to community). A potentially more mature example can be seen in SICP, which has implemented a single patient record accessible to the professionals of the case management multidisciplinary team and the emergency medicine professionals. Their ultimate goal is for the platform to be accessible by primary, secondary, and community care organizations in the Salford area. In a minority of the programs (eg, ON and PCSS), data transfer across various IT platforms of providers are manually performed by program administrators. All in all, most ICC programs indicate the determinant positive role of the existing regional digital health tools for health information exchange across health care tiers, which facilitates information sharing among heterogeneous providers, as seen in this example from the Proactive Primary Care Approach for Frail Elderly (U-PROFIT) program in the Netherlands:

We are working with a vulnerable population, frail in general, and it is important for them to avoid going from one place to another and visiting different service providers and collecting different forms [...] or duplicate papers because you have to present this paper here and this same paper over there [...] I think this is an important progress for the population

IP04_1—U-PROFIT

A step forward in terms of organizational interoperability, the computerization of health and social care records via a *shared EMR* among health care tiers, is at the heart of some ICC programs, such as the GeroS program. In line with organizational interoperability, the Care Chain Frail Elderly (CCFE) program focuses on structuring care and stimulating communication between all chain partners in primary care at various access levels with one another, thanks to an additional digital health tool (Care2U) that is used on top of the existing information systems to access the individual care plan and exchange information. However, although there has been much effort by the governments of these countries to have a shared EMR in place, this has not yet been fully successful, mostly due to data privacy issues. Last but not least, the AISBE program aims to consolidate a *shared case management system* [36] on top of the existing regional shared EMR, aiming to support the regional deployment of adaptive case management processes.

#### Digital Transformation Policies (Macro)

As all 17 ICC programs have at least partial implementation of EMR, all national and regional policies aim to expand the implementation of *EMR* in the future. However, in most program countries, the use and scope of digital health tools depends on *several initiatives at program level*, which serve as pilot sites for the nation and region wide rollout of digital health tools. This is the case, for example, in the Austrian programs (HNT and Sociomedical Centre Liebenau [SMC]), which are part of the *electronic health files*, the most comprehensive eHealth initiative in Austria. Our research has only been able to identify *national and regional strategic plans* for deployment of eHealth in Croatia, Spain, Hungary, the Netherlands, Norway, and the United Kingdom. The largest digital transformation policy being the Whole System Demonstrator pilots in the United Kingdom, which is a strategy proposed by the Department of Health in England to focus on health and social care for people with long-term needs, emphasizing the use of advanced assistive technologies including telehealth and telecare. It has demonstrated a slight reduction in mortality and emergency admission rates but was not demonstrated to be more cost effective than usual care [37,38].

Under the auspices of the Norwegian Directorate of Health, a *Care Journal* has been recently established for all citizens (voluntary); this is an electronic tool comprising selected and important health data that are accessible for the citizen and health personnel for the whole health care sector in Norway (including the 2 programs analyzed in this paper). Another example is the Catalan Health Plan [39], which prioritizes the improvement and transformation of the health system and health care organization through the intensive introduction of emerging digital health technologies.

### Information and Research

As summarized in the Multimedia Appendix 3, the overarching analysis provided the following valuable insights on the assessment strategies of the 17 technology-enabled ICC programs.

#### Evaluation Strategies (Micro)

This research has shown that in some ICC programs, no comprehensive evaluation has been carried out so far (SMC, Palliative Care System, ON, and Learning network), but is *planned* to be performed.

However, most ICC programs have been subject to *partial monitoring and/or preliminary evaluation* (ie, HNT, GeroS, Palliative Care Consulting Service, CCFE, BSiN, MAR, SICP, and SSSP), involving mainly descriptive data analysis over well-defined outcome measures of interest or key performance indicators. Specifically, the SSSP program includes:

Number of bed days, average length of stay, 30 day readmission, avoidable emergency admissions, precautionary emergency admissions, patients admitted multiple times, excess bed days, avoidable A&E attendances, confidence to my own health, received enough support to help self-managed long-term conditions, have a written care plan, care plan regularly reviewed, patient access to GP and nurse, online services, GP referrals, mental wellbeing, the Warwick-Edinburgh Mental Wellbeing scale, patient activation measure [PAM], patient satisfaction experience, and number of contacts made.

IP03_2—SSSP

Only some programs report *full scientific assessment* (ie, Casaplus [40], Gesundes Kinzigtal [41,42], U-PROFIT [43,44], and AISBE [45-47]). These programs have been evaluated using randomized controlled trials as well as pre-post evaluation with propensity score matching methods, following the Triple Aim outcomes [48,49]:

...was the number of hospital admissions reduced? How did they experience the effects of care (the insured person, the environment, the relatives)? Were the per capita costs reduced?

IP04_1—Casaplus

#### Risk Assessment Strategies (Meso)

Patient management purely based on *clinical criteria* (professional training, knowledge, instinct, and experience) *or combined with rules-based clinical management* [50] (thresholds for certain parameters defining pre-established decision criteria) constitutes current health professional practice in most ICC programs.

In contrast, the regular use of *subject-specific predictive modelling* tools for clinical decision support (predictive modelling establishing relationships between sets of variables and outcomes generated using statistical or machine learning tools) is still in its infancy, despite the fact that it seems a natural step toward customization of care to patient’s needs. Clinical predictive modelling tools are only reported to be used in some ICC programs (ie, SICP, SSSP, U-PROFIT, Gesundes Kinzigtal, and Casaplus) *for individual risk assessment* (which can be considered within the micro level). Within the U-PROFIT program, available data in the general practitioner EMR system are used by the U-PRIM software to screen frail patients of 60 years and older in every participating practice [51]. SICP and SSSP programs in the United Kingdom use a well-known patient-level risk predictive tool, PARS [52] and the Combined Predictive Model [53], to identify those patients that require the most care and support and to assess the risk of patients having unplanned hospital admissions within a 12-month period. It is used to some extent, but more trust is placed in clinical judgement in many cases:

...so we’d looked at some of the higher risk patients that were identified by the Combined Predictive Model and PARS (Patient at Risk Score) exactly the same, because I’ve done that before for the unscheduled care, and we looked at that; and what you find is the high risk people that are identified by this risk stratification models that are promoted nationally, is that the only data that’s easy to count is the hospital data, is the Hospital Episode Statistics of your hospital episodic statistics and stuff.

IP02_1—SICP

Similarly, Casaplus uses a clinical predictive modelling tool to identify patients in high risk for hospital admissions within the next 12 months.

The use of clinical predictive modelling tools for *population-based risk assessment* is only reported in BSA and AISBE (Catalonia, ES). Since 2011, the Catalan Health Surveillance system collects detailed information on health care usage for the entire population of Catalonia [54], the region in which AISBE and BSA operate. It includes information on hospitalization, primary care visits, emergency department visits, skilled nursing facilities, palliative care and the mental health services, information on pharmacy prescription and expenditure, and a registry on the billing record also encompassing outpatient visits to specialists, home hospitalization, medical transportation (urgent and nonurgent), ambulatory rehabilitation, respiratory therapies, and dialysis. This information is used for provider payment purposes. Also, external audits are performed periodically to ensure the quality and reliability of the data. The Catalan Health Surveillance System is used to update, on a 6-month basis, the regional population-based health risk assessment tool, (the Adjusted Morbidity Groups) that generates the health risk strata pyramid of the general population of Catalonia [13,14].

Furthermore, AISBE is adopting a holistic approach that fosters inclusion of covariates from multilevel data sources, namely *Multilevel Predictive Modelling*: (1) clinical, (2) informal care; (3) biological research; and (4) outcomes from population-health risk predictive modelling (eg, the Adjusted Morbidity Groups), resulting in enhanced patient-based stratification and optimization of service selection. This approach aims to pave the way toward personalized medicine, provided that access to the multilevel data sources is granted. However, most legal frameworks on data privacy of the 17 ICC programs depend on the ongoing implementation of the European Union Data Protection Directive 95/46/EC to make the concept of multilevel predictive modelling operational.

#### Research and Innovation Policies (Macro)

A majority of the 17 ICC programs are part of *incipient research and innovation initiatives* constantly being implemented in practice, both bottom-up and top-down, using several, sometimes consecutive, project-budgets but without sustainable structural funding.

However, Croatian, Dutch, German, Norway, and Spanish programs are aligned with *consolidated research and innovation programs* at state and regional level. For example, the Gesundes Kinzigtal program in Germany has been extensively evaluated in terms of prevalence of multimorbidity, polypharmacy, proportion of generic drugs, prevalence of problematic drug prescriptions, prevalence of fractures among patients diagnosed with osteoporosis, quality of services, and overall health care costs [42]. Another example is the Research Council of Norway, commissioned by the Ministry of Health and Care Services to carry out a research-based evaluation of the Co-ordination Reform. The Research Council has conducted a research program tailored at integrated care from 2012 to 2015.

Furthermore, the *strong co-ordination* of most programs from Norway, the Netherlands, Hungary, Spain, Croatia, and Germany *with different European research and innovation initiatives* under the umbrella of H2020 [55], EIP-AHA [56], EIT Health [57], and/or RIS3 [58], as well as other specific research and innovation actions, should contribute to cross-fertilization among health care, research, and innovation.

## Discussion

### Principal Findings

The overarching analysis allowed us to assess the use of digital health tools to support the care process in the 17 ICC programs on the 2 specific aspects analyzed: *Technologies & Medical* products and *Information & Research*. As most of the ICC programs are pilot experiences in terms of nation and region wide rollout of digital health tools, this analysis was useful to learn from them regarding requirements for a successful large-scale implementation elsewhere.

Acknowledging that the 17 ICC programs are highly heterogeneous regarding the use and impact of digital health tools, the main findings are summarized below.

#### Electronic Medical Records

Each program studied shows at least partial implementation of EMR, and all of them have plans in place for a future mature implementation of EMR.

#### Personal Health Records

The use of personal health records to support telemonitoring services for patient self-management is not in place in most ICC programs, for which telephone is still the dominant means of communication.

#### Health Information Exchange Platforms

Most programs reported on the potential of secure health information exchange across providers to facilitate organizational interoperability for deployment of ICC by facilitating information sharing among heterogeneous providers and avoid generating additional burden of double-registration to health professionals. However, the maturity of implementation is currently rather poor. Moreover, the need for technological tools, on top of health information exchange platforms, supporting collaborative work across health care tiers to foster implementation of shared case management [36,59] was stressed by programs like AISBE.

#### Digital Transformation Policies

The overarching analysis highlighted the lack of well-defined macro-level policies, with effective operational implementation plans, in the health care systems in which most ICC programs operate. Often, the use and scope of digital health tools depends on local or regional initiatives of individual providers involved in the ICC programs.

#### Health Data Analytics and Evaluation Strategies

Most programs systematically collect well-defined outcome measures to feed program-specific evaluation strategies, ranging from descriptive data analysis, comparison of trial and control groups, as well as pre- and postmeasuring. Still, some ICC programs recognize barriers for assessment such as a lack of financing, poor research capacity, concerns on data security, and misuse of data. This study clearly shows the need for formulation of structured and comprehensive evaluation and monitoring strategies for ICC including formulation of key performance indicators extensively shared across countries. Moreover, the Quadruple Aim approach [60,61] (ie, the Triple Aim approach plus the health care professionals experience) should serve to standardize the evaluation across European Union sites.

#### Health Risk Assessment

Just a few of the ICC programs report on the use of clinical predictive modelling tools, and even less ICC programs claim the use of population-based health-risk prediction tools.

#### Research and Innovation Policies

The majority of the 17 ICC programs (either bottom-up or top-down initiatives) are often based on project-specific budgets without well-defined, operational policies and, consequently, without sustainable structural funding. Implementing the above technological innovations frequently requires hardware and software upgrades. The costs of initial rollout and training of staff also need to be considered and weighed against the likely benefits.

We acknowledge some limitation in our study such as the inherent limitations of the methodological approach adopted. Also, as the study conclusions relate only to programs based in Europe, worldwide representativeness of the study results cannot be assumed.

### Comparison With Previous Work and Future Directions

A recent report by the European Union on Integrated Care maturity [62], including the evaluation of Information and eHealth tools, concluded that the level of maturity in Germany, Denmark, Belgium, Italy, Spain, Greece, Sweden, and Iceland scored higher in comparison to their peers in Estonia, the Netherlands, Poland, and Bulgaria. The most mature countries are Denmark, followed by Spain, Germany, and Iceland at the same level. Finally, Sweden, Belgium, Italy, and Greece were in intermediate level. This is line with our findings, except for the 3 case studies in the Netherlands, which in our study were more mature than Dates et al [62] suggests. It is of note that differences between the study by Dates et al [62] and this study might be explained by using different tools as well as the selection of 3 promising cases in the Netherlands which actually use eHealth tools. The European Union on Integrated Care maturity report applies an interactive tool developed by SIROCCO (Scaling Integrated Care in Context) to assess the maturity of ICC programs on a global level using different aspects (one of them being eHealth); whereas in our study, we explicitly focus on different aspects of digital health tools and assessment strategies solely. Also, the previous report is based on a review of the literature on integrated care policies and strategies, whereas our study adds the point of view from the different stakeholders directly involved in generation and implementation of these policies and strategies.

Integrated care programs for chronic patients involve complex interventions for heterogeneous populations; therefore, proper articulation of digital health tools and the different components of the evaluation process are still unmet needs that markedly hinder comparability and scale-up. The overarching analysis of the 17 ICC programs conducted in this study allowed us to identify the following potential areas for future developments:

*Refinement of assessment methodologies* of large-scale deployment and adoption of ICC programs, likely based on implementation research approaches [63-65], are needed. We understand that assessment should adopt the classical three-dimensional approach including outcomes, processes, and structures [66]. Moreover, usual health outcome variables (ie, mortality, hospital readmissions, etc.) should be ideally expanded [67] considering the Quadruple Aim approach [60,61]. The approach requires the collection of patient-reported outcomes and experience data (PROMS and PREMS) on a regular basis.

*The concept of adaptive case management* explored in AISBE [36,68] should be made operational. Conventional health information systems rely on the management of clinical episodes with a disease-oriented approach and only very rarely incorporate the required process logics to support continuity of care with a patient-centered approach.

*Dynamic health-risk assessment* taking into consideration both service commissioning (*population-based health-risk predictive modelling*) and subject-specific service selection involving optimal patient allocation in the health system (*individual health-risk predictive modelling supporting decision support*) should be addressed to improve outcomes [69-71]. Ultimately, the application of holistic strategies for subject-specific risk prediction and stratification that incorporates multilevel determinants of health (eg, socioeconomic, lifestyle, behavioral, clinical, physiological, cellular, and *omics* information) emerges as a high priority goal to properly pave the way toward personalized medicine for complex chronic patients [72]. Enhanced clinical predictive modelling, personalized diagnostic and treatment tools can contribute to the acceleration of transfer of scientific evidence to practice.

*Development of pragmatic trials that incorporate real-life evidence* from multilevel determinants of health may require implementation strategies, ideally using cloud computing environments, tackling privacy and regulatory constraints [23,72]. Currently, the articulation of the main technical building blocks, that is, multilevel biomedical data integration, tools for clinical predictive modelling in the cloud and High-Performance Computing, as one integrated system is yet a largely unmet potential.

### Conclusions

This overarching analysis informs the current implementation status of digital health tools for management of multimorbidity in the 17 promising ICC programs selected in SELFIE. Notwithstanding the heterogeneity of the results, most studied programs are progressively evolving their supporting digital health tools from pilot prototypes to full scale-up at regional and national level. However, the majority of programs have not yet undergone full evaluation and assessment strategies. Future directions which can enable of digital transformation are based on innovation at micro and meso level with full support from the macro level. Some strategic areas that can help toward this end are the following: (1) implementation of research strategies; (2) explore an adaptive case management approach; (3) further developments of health risk assessment; and (4) holistic implementation strategies using future, regulatory compliant, cloud computing environments.

## Appendix

Multimedia Appendix 1 Essential and additional criteria for preliminary selection of Sustainable intEgrated care modeLs for multimorbidity: delivery, Financing, and performancE (SELFIE) programs.

Multimedia Appendix 2 Stakeholders interviews per country.

Multimedia Appendix 3 Summary of the overarching analysis of the 17 selected integrated chronic care programs.

## Abbreviations

AISBE: Area Integral de Salut Barcelona-Esquerra
BSA: Badalona Serveis Assistencials
BSiN: Better together in Amsterdam North
CCFE: Care Chain Frail Elderly
EMR: Electronic Medical Record
HNT: Health Network Tennengau (Gesundheitsnetzwerk Tennengau)
ICC: Integrated Chronic Care
MAR: Medically Assisted Rehabilitation
ON: OnkoNetwork
SELFIE: Sustainable intEgrated care modeLs for multimorbidity: delivery, Financing, and performancE
SICP: Salford Integrated Care Program
SMC: Sociomedical Centre Liebenau (Sozialmedizinisches Zentrum Liebenau)
SSSP: South Somerset Symphony Program
U-PROFIT: Proactive Primary Care Approach for Frail Elderly
