# Supplementary material for: Digital Health Transformation of Integrated Care in Europe: Overarching Analysis of 17 Integrated Care Programs
Source: J Med Internet Res. 2019 Sep 26;21(9):e14956. doi: 10.2196/14956 (PMC6794072; doi:10.2196/14956)
Supplement: Multimedia Appendix 1 [file jmir_v21i8e14956_app1.pdf]

1. Multimedia Appendix 1

Supplementary Table 1. Essential and additional criteria

Essential
Multimorbidity (broad definition incl. vulnerable groups)
Multidisciplinary integrated care
Innovative in country's context (e.g. intersectoral integration, population health management)
Outcome data available or collectable for each of the triple aims of population health, care experience and costs
Ongoing for at least the next 2 years
**Additional**
Does the patient have an active role?
Is the programme goal-oriented?
Does the programme ensure continuity of care?
Are informal caregivers actively involved?
Was the programme a bottom-up initiative?
Can the programme be scaled-up and is it transferable?
Variability across selected programmes

Supplementary Table 2. Preliminary selection of programmes

AT	Health Network Tennengau (Gesundheitsnetzwerk Tennengau)<br>Sociomedical Centre Liebenau (Sozialmedizinisches Zentrum Liebenau)
HR	GeroS System<br>Palliative Care System
DE	Casaplus<br>Gesundes Kinzigtal
HU	Onconetwork<br>Palliative Care Consulting Service (Mobile) Team
NO	Learning network<br>Medically Assisted Rehabilitation (MAR) Bergen
ES	Badalona Serveis Assistencials (BSA)<br>Barcelona Esquerre (AISBE)
NL	Better together in Amsterdam North (BSiN)<br>Proactive Primary Care Approach for Frail Elderly (U-PROFIT)<br>Care Chain Frail Elderly (previously called KOMPLEET)*
UK	South Somerset Symphony Programme<br>Salford – Salford Integrated Care Programme (SICP)/ Salford Together

* Due to additional internal funding, three projects are included in The Netherlands.
