# Supplementary material for: Digital Health Transformation of Integrated Care in Europe: Overarching Analysis of 17 Integrated Care Programs
Source: J Med Internet Res. 2019 Sep 26;21(9):e14956. doi: 10.2196/14956 (PMC6794072; doi:10.2196/14956)
Supplement: Multimedia Appendix 2 [file jmir_v21i8e14956_app2.pdf]

Multimedia Appendix 2: Stakeholders interviews per country

Country	Managers	Initiators	Payers	Physicians	Other medical staff	Informal caregivers	Patients	Other
Austria	3	3	5	5	11	5	3	1
Croatia	3	3	3	6	9	3	3	1
Germany	6	3	3	3	4	1	4	0
Hungary	3	3	2	8	12	4	0	0
The Netherlands	6	6	5	3	9	3	4	2
Norway	2	2	2	4	7	2	1	3
Spain	5	1	1	4	7	3	1	2
United Kingdom	4	4	2	4	7	2	2	0
**TOTAL**	**32**	**25**	**23**	**37**	**66**	**23**	**18**	**9**
