# Supplementary material for: Digital Health Transformation of Integrated Care in Europe: Overarching Analysis of 17 Integrated Care Programs
Source: J Med Internet Res. 2019 Sep 26;21(9):e14956. doi: 10.2196/14956 (PMC6794072; doi:10.2196/14956)
Supplement: Multimedia Appendix 3 [file jmir_v21i8e14956_app3.pdf]

Multimedia Appendix 3: Summary of the overarching analysis of the 17 selected integrated chronic care programs.

ICC[a] type	Country	Name of ICC program	Macro summary of ICC program [1]	Technologies and medical products	Information and research
Population Health Management	Austria (AU)	Health Network Tennengau (HNT)	Bottom-up network comprises social and health service providers and voluntary organizations [2].	Secure data network between the hospital and approximately 100 regional GP[b]. Applications for patients are not available.	Descriptive data analysis.
	Germany (DE)	Gesundes Kinzigtal (GK)	Population-based approach that organizes care across all health service sectors and indications [3].	System-wide electronic health record for health provider and digital benchmark information.	Triple aim external and internal scientific evaluation.
	Spain (ES)	Área Integral de Salut, Barcelona Esquerra (AISBE)	Care co-ordination among different providers and care levels in 1 of the 4 health care sectors of the city of Barcelona [4].	Shared electronic health records at regional level and Patient Gateway. Self-management tools for patients and adaptive case	Continuous assessment through the Catalan Health Surveillance System. Population-based risk assessment tool (the

				management tools for professionals.	Adjusted Morbidity Groups).
	United Kingdom (UK)	Salford Integrated Care Program (SICP) and Salford Together	Originally aimed at the elderly (>65 years) and consists of case management of high-risk patients with support of community assets and center of contact for prevention and co-ordination [5].	Use of population risk-stratification tool and dashboard. Partial introduction of shared electronic medical records. Some disease-specific use of telehealth and other technology innovations.	Continuous monitoring of process indicators and rigorous academic evaluation working with the local university and NHS[c] England.
	United Kingdom (UK)	South Somerset Symphony Program (SSSP)	Aimed at multimorbidity and consists of GPs located in a hospital hub individually managing the most complex patients and colocation of health coaches in primary care to assist with disease self-management and prevention [5].	Some use of population risk-stratification tool for selecting complex patients. Problems introducing shared health records for professional access. Some patient use of new web-based record, but	Continuous monitoring of key outcome set. Working with Universities and NHS England for robust external evaluation.

				uptake poor. Some use of telehealth.	
Frail Elderly	Croatia (HR)	GeroS	Integrated care model for geriatric patients with multimorbidity [6].	Electronic health and social care records via a central database. Not all modules fully integrated yet.	A monitoring system has been introduced but a quality assurance system has not yet been established.
	Germany (DE)	Casaplus	Case management program for elderly (>55 years) with multiple chronic conditions and at high risk for hospital admission(s) within the next 12 months [3].	Web-based platform to support regular communication between case managers and nursing professionals.	Continuous external and internal scientific evaluation.
	The Netherlands (NL)	Care Chain Frail Elderly (CCFE)	Targets vulnerable older persons living at home with complex care needs [7].	Secured ICT[d] infrastructure (Care2U) on which individual care plans are posted and professionals from different disciplines can share	Extraction of quality indicators from Care2U for routine monitoring of the process plus small-scale qualitative

				information. Different professionals have different levels of access to data. Data entered are automatically transferred to the GPs' information systems, but not to the information systems of the other professionals.	evaluations by the insurer.
	Norway (NO)	Learning networks for whole, co-ordinated and safe pathways (LN)	Program focusing on older persons enrolled in home nursing service or short-term stay in nursing home [8].	Electronic white boards showing patient status.	No scientific evaluation conducted so far.
	The Netherlands (NL)	Proactive Primary Care Approach for Frail Elderly (U-PROFIT)	Nurse-led intervention for frail elderly (>60 years) living at home [7].	Specific software is used to screen for frail elderly in the electronic medical records of the GPs. Once identified the	Extensively evaluated, including randomized controlled trials. Mixture of stand-alone

				second screening-step includes a frailty questionnaire and the third step includes a home visit. Many GP's use a care chain information system that is not compatible with information systems of other organizations.	data-collection and data extraction from electronic medical records.
	Spain (ES)	Badalona Serveis Assistencials (BSA)	Provides healthcare and social support services with 24-7-365 emergency support [4].	Shared electronic health records at regional level and Patient Gateway. Telemonitoring services at pilot level.	Continuous assessment through the Catalan Health Surveillance System. Population-based risk assessment tool (the Adjusted Morbidity Groups).
Palliative care and oncology	Croatia (HR)	Palliative Care System (PCS)	Integrated care program for	County-specific software to identify and	Use of questionnaires to evaluate the

			palliative care patients [6].	monitor palliative patients. No common IT system at national level.	extent to which certain elements of palliative care services have been established.
	Hungary (HU)	Palliative Care Consult Service (PCCS)	Supports patient pathway management across providers (eg, between secondary care to homecare) [9].	Hospital electronic referral system to support consultation requests and keep record of the electronic documentation.	Assessment of pain and performance status and professional satisfaction surveys.
	Hungary (HU)	OnkoNetwork (ON)	Local initiative to improve clinical outcomes via timely access to quality assured and unfragmented health care [9].	IT system for patient path monitoring and management. Interoperability with other IT systems is a challenge.	No outcome analysis has been conducted so far. Limited data on patient experience.
Patients with problems in multiple domains of life besides their health	Austria (AU)	Sociomedical Centre Liebenau (SMC)	Bottom-up model providing health and social care predominantly to vulnerable and disadvantaged groups [2].	Electronic data gathering and processing system. Applications for patients are viewed critically.	No comprehensive evaluation has been carried out so far.

	The Netherlands (NL)	Better Together in Amsterdam North (BSiN)	Targets persons with low self-sufficiency and complex needs in multiple life domains [7].	Easy to use but stand-alone web-based ICT support system that is not compatible with the ICT systems of the organizations involved in BSiN. This system is used for enrollment, triage and case management.	Preliminary short-term (6-month) assessment of self-sufficiency.
	Norway (NO)	Medically Assisted Rehabilitation (MAR) Bergen	Treatment program for opioid addiction administered by the Bergen hospital enterprise [8]	Electronic medical record for evaluation. Problematic sharing of medical records by specialists and social services.	Research is ongoing by different groups with some outcomes for the relevant patient groups.

[a]ICC: integrated chronic care.

[b]GP: general practitioner.

[c]NHS: National Health Service.

[d]ICT: information and communication technologies

References

1. SELFIE. SELFIE case studies. 2018.https://www.selfie2020.eu/publications/

2. SELFIE. Austria: Case studies. 2018.https://www.selfie2020.eu/wp-content/uploads/2018/06/SELFIE_WP-2_Final-report_Austria.pdf
3. SELFIE. GERMANY: case studies. 2018.https://www.selfie2020.eu/wp-content/uploads/2016/12/SELFIE_WP2_Germany_Final-thick-descriptions.pdf
4. SELFIE. SPAIN: case studies. 2018.https://www.selfie2020.eu/wp-content/uploads/2016/12/SELFIE_WP2_Spain_Final-thick-descriptions.pdf
5. SELFIE. UNITED KINGDOM: case studies. 2018.https://www.selfie2020.eu/wp-content/uploads/2016/12/SELFIE_WP2_UK_Final-thick-descriptions.pdf
6. SELFIE. CROATIA: case studies. 2018.https://www.selfie2020.eu/wp-content/uploads/2016/12/SELFIE_WP2_Croatia_Final-thick-descriptions.pdf
7. SELFIE. NETHERLANDS: case studies. 2018.https://www.selfie2020.eu/wp-content/uploads/2016/12/SELFIE_WP2_Netherlands_Final-thick-descriptions.pdf
8. SELFIE. NORWAY: case studies. 2018.https://www.selfie2020.eu/wp-content/uploads/2016/12/SELFIE_WP2_Norway_Final-thick-descriptions.pdf
9. SELFIE. HUNGARY: case studies. 2018.
